# Funktionelles Outcome nach konservativer im Vergleich zu operativer Therapie von 111 Mittelfußfrakturen

**DOI:** 10.1007/s00113-021-01006-6

**Published:** 2021-05-22

**Authors:** Patrick Pflüger, Michael Zyskowski, Christopher Völk, Chlodwig Kirchhoff, Peter Biberthaler, Moritz Crönlein

**Affiliations:** Klinik und Poliklinik für Unfallchirurgie, Klinikum rechts der Isar, Technische Universität München, München, Deutschland

**Keywords:** Fuß, Frakturen, ORIF, Konservative Therapie, Selbstberichteter patientenbasierter Ergebnisfragebogen, Foot, Fractures, ORIF, Conservative therapy, Patient reported outcome

## Abstract

**Hintergrund:**

Mittelfußfrakturen gehören zu einer der häufigsten Verletzungen des Fußes und treten v. a. bei Patienten zwischen dem 40. und 50. Lebensjahr auf. Insbesondere die Verletzung mehrerer Mittelfußknochen kann zu bleibenden Einschränkungen führen, und daher war das Ziel dieser Studie, das funktionelle Outcome von Mittelfußfrakturen mittels eines validierten selbstberichteten patientenbasierten Ergebnisfragebogens zu untersuchen.

**Material und Methoden:**

Im Zeitraum von 2003 bis 2015 wurden alle Patienten mit einer Mittelfußfraktur mit konservativer sowie operativer Behandlung in diese retrospektive Kohortenstudie eingeschlossen. Es wurden demografische Daten, Art der Fraktur (AO-Klassifikation), Behandlung, Reoperationsrate sowie das funktionelle Ergebnis mittels Foot and Ankle Outcome Score (FAOS) erfasst. Der Mann-Whitney-U-Test und Exakte Fisher-Test wurden bei der statistischen Analyse eingesetzt.

**Ergebnisse:**

Insgesamt wurden in diese Studie 111 Patienten mit 81 isolierten und 30 multiplen Mittelfußfrakturen eingeschlossen. Das Durchschnittsalter der Patienten war 45 ± 15,2 Jahre mit insgesamt 48 Männern (43 %) und 63 Frauen (57 %). Patienten mit isolierter Mittelfußfraktur hatten einen FAOS von 88 ± 17,1. Die Patienten mit multiplen Mittelfußfrakturen erzielten einen FAOS von 78 ± 17,7 (*p* = 0,046). In der Gruppe der isolierten Mittelfußfrakturen wurden 43 Patienten (53 %) operativ behandelt. Hiervon zeigten 36 Patienten eine C‑Fraktur (84 %). In der Gruppe der multiplen Mittelfußfrakturen wurden 16 Patienten (53 %) operativ behandelt.

**Diskussion:**

Das funktionelle Outcome nach isolierten Mittelfußfrakturen ist sowohl nach operativer als auch konservativer Therapie gut bis sehr gut. Einfache Frakturen lassen sich erfolgreich konservativ und komplexe, mehrfragmentäre Frakturen operativ behandeln. Bei Frakturen von mehr als einem Mittelfußknochen ist das Ergebnis signifikant schlechter, und es bleiben vom Patienten berichtete Einschränkungen zurück.

## Abstract (en)

**Background:**

Fractures of the metatarsal bones are common injuries of the foot and particularly occur in patients aged 40–50 years. Especially multiple metatarsal fractures can lead to permanent limitations. Therefore, the aim of this study was to investigate the functional outcome of metatarsal fractures after conservative and surgical treatment using a validated self-reported patient-based outcome questionnaire.

**Material and methods:**

All patients suffering from metatarsal fractures between 2003 and 2015 were enrolled in this retrospective analysis. The following data were collected: demographic data, AO classification, treatment, reoperation rate and the foot and ankle outcome score (FAOS). For outcome analysis, the nonparametric Mann-Whitney U‑test and Fisher’s exact test were performed.

**Results:**

In total the functional outcome of 111 patients with metatarsal fractures were analyzed, 81 patients suffered of an isolated metatarsal fracture and 30 of multiple fractures. The mean age of the patients was 45 ± 15.2 years with a total of 48 men (43%) and 63 women (57%). Patients with an isolated metatarsal fracture had an FAOS of 88 ± 17.1, while patients with multiple metatarsal fractures achieved an FAOS of 78 ± 17.7 (*p* = 0.046). In the group of isolated metatarsal fractures 43 patients (53%) were surgically treated and of these 36 patients showed a type C fracture (84%). In the group of multiple metatarsal fractures 16 patients (53%) underwent operative treatment.

**Conclusion:**

Overall, the functional outcome of isolated metatarsal fractures following operative as well as conservative treatment is good to very good. Simple fractures can be successfully treated conservatively and complex multifragment fractures can be safely managed surgically. If more than one metatarsal bone is fractured, the functional outcome is significantly worse with patients reporting lasting limitations involving the range of motion and stiffness.

## Einleitung

Frakturen des Mittelfußes zählen zu den häufigsten Verletzungen der unteren Extremität. Einfache Mittelfußfrakturen zeigen unter konservativer Therapie sehr gute Ergebnisse. Bei der Beteiligung von mehreren Mittelfußknochen kann es jedoch zu bleibenden Einschränkungen mit chronischen Schmerzen kommen. In der Literatur finden sich nur wenige Studien, welche sich mit dem Ergebnis nach konservativer sowie operativer Therapie beschäftigt haben. Deshalb haben wir das Outcome nach Mittelfußfraktur mittels eines selbstberichteten patientenbasierten Ergebnisfragebogens, dem Foot and Ankle Outcome Score (FAOS), untersucht.

## Hintergrund und Fragestellung

Mittelfußfrakturen gehören mit einer Inzidenz von 75 pro 100.000 Personen und Jahr zu einer der häufigsten Verletzungen des Fußes [9, 10, 16]. Das mittlere Alter der Patienten ist um das 40. Lebensjahr, wobei v. a. im höheren Alter überproportional mehr Frauen betroffen sind [9, 16]. Bei der Mehrzahl handelt es sich um einzelne Mittelfußfrakturen, welche am häufigsten den 5. Mittelfußknochen betreffen [9, 16]. Frakturen mehrerer Mittelfußknochen sind meist unmittelbar nebeneinander lokalisiert und mit Begleitverletzungen vergesellschaftet [16]. Insbesondere bei komplexen Mittelfußfrakturen, welche die Basis betreffen, können Verletzungen des Tarsometatarsalgelenks (Lisfranc-Gelenk) auftreten [12, 23]. Verletzungen des Lisfranc-Gelenks sind selten, können jedoch zu dauerhaften Einschränkungen und Schmerzen führen [12, 23]. Daher ist für eine genaue Klassifikation der Frakturmorphologie und möglicher Begleitverletzungen insbesondere im Falle mehrerer Mittelfußfrakturen eine Computertomographie indiziert [7, 12].

Einfache, nichtdislozierte Mittelfußfrakturen können konservativ behandelt werden und zeigen sehr gute funktionelle Ergebnisse [9, 25]. Bei dislozierten Schaft- oder Halsfrakturen kann mittels einer minimal-invasiven K‑Draht-Osteosynthese eine anatomische Reposition erzielt werden [19]. Diese weichteilschonende Technik führt zu guten klinischen Ergebnissen [24]. Mehrfragmentäre, dislozierte Frakturen mit Gelenkbeteiligung erfordern eine offene Reposition und interne Fixierung mittels Platten‑/Schraubenosteosynthese [18]. In diesen Fällen zeigt die Versorgung mittels Plattenosteosynthese gute Ergebnisse mit knöcherner Konsolidierung und zeitnaher Vollbelastung [8]. Komplexe Verletzungen des Mittelfußes mit Trümmerfrakturen und signifikantem Weichteilschaden können jedoch auch zu dauerhaften Beschwerden führen [2].

Betrachtet man die Literatur, so existieren wenige Studien, welche die Behandlung und das funktionelle Ergebnis von Frakturen des 1. bis 4. Mittelfußknochens analysieren [7, 20]. Darüber hinaus sind bei diesen retrospektiven Studien, welche sich mit dem operativen Ergebnis von Mittelfußfrakturen beschäftigten, nur weniger als 50 Patienten untersucht worden [8, 13]. Es existiert lediglich eine größere Studie mit mehr als 300 Patienten von Cakir et al., die das funktionelle Ergebnis nach konservativer Behandlung von Mittelfußfrakturen untersuchten [9]. Daher war es das Ziel dieser retrospektiven Arbeit, das funktionelle Outcome nach konservativer und operativer Behandlung von Mittelfußfrakturen zu analysieren.

## Material und Methoden

Patienten mit einer Mittelfußfraktur, welche im Zeitraum von 2003 bis 2015 in der Klinik und Poliklinik für Unfallchirurgie der Technischen Universität München behandelt wurden, wurden nach positivem Ethikvotum (Nr.: 409/15 S, Technische Universität München) retrospektiv nachuntersucht. Die Einschlusskriterien waren: Patienten mit einer oder mehreren geschlossenen Mittelfußfrakturen (ohne isolierte MT-V-Basis-Frakturen nach Lawrence und Botte), Alter > 15 Jahre und Einwilligungsfähigkeit. Ausschlusskriterien waren: Lisfranc-Gelenk-Luxation, Patienten mit pathologischer Fraktur, Zustand nach operativer Versorgung ex domo, Drogenabhängigkeit und gesetzliche Betreuung. Sowohl konservativ als auch operativ behandelte Patienten wurden in die Analyse einbezogen.

Entsprechend den AO-Leitlinien erfolgte eine operative Versorgung bei Patienten mit signifikantem Rotationsfehler, relevanter Verkürzung > 5 mm, Gelenkstufe > 2 mm, Achsabweichung > 20°, multiplen intraartikulären Frakturen, Dislokation im Metatarsophalangealgelenk in Kombination mit einer intraartikulären Fraktur des 1. Mittelfußknochens und im Falle von dislozierten Mehrfragmentfrakturen. Voraussetzungen für die operative Versorgung waren eine entsprechende gute Weichteilsituation und Operabilität des Patienten.

Das funktionelle Ergebnis wurde mittels Foot and Ankle Outcome Score (FAOS) erfasst. Der FAOS ist ein selbstberichteter patientenbasierter Ergebnisfragebogen, welcher aus 42 Items besteht (Bereich: 0 bis 100 Punkte). Die deutsche Version des FAOS ist ein validiertes und reliables Messinstrument für Fuß- und Sprunggelenkpathologien [22].

Nach erfolgter stationärer oder ambulanter Behandlung wurden die Patienten, welche die Einschlusskriterien erfüllten, postalisch zur Teilnahme und zum Ausfüllen des FAOS eingeladen.

Die eingeschlossenen Mittelfußfrakturen wurden anhand der Röntgenbilder nach AO/OTA klassifiziert [14].

Allgemeine Daten wie Alter, Geschlecht, betroffene Seite, Nachbeobachtungszeitraum, Zeit zwischen Fraktur und Operation sowie Reoperationsrate wurden erfasst. Die operative Therapie umfasste die offene Reposition und interne Fixation mittels Plattenosteosynthese, Schrauben- oder minimal-invasiver K‑Draht-Osteosynthese. Die Patienten erhielten postoperativ nach Konsolidierung der Weichteile eine kurze Unterschenkelfußorthese für 6 Wochen mit einer vorgegebenen Teilbelastung von 15 kg. Es erfolgten radiologische Kontrollen 6 Wochen, 3 Monate und ein Jahr postoperativ.

Die konservative Therapie beinhaltete eine Teilbelastung mit 15 kg für 6 Wochen an Unterarmgehstützen in einer kurzen Unterschenkelfußorthese. Radiologische Kontrollen erfolgten 4, 7, 11 Tage und 6 Wochen nach dem Trauma. Bei regelrechtem radiologischen Befund nach 6 Wochen durften die Patienten zur Vollbelastung übergehen und die Unterschenkelfußorthese ablegen.

### Statistische Auswertung

Die Daten sind als Median ± Standardabweichung (SD) dargestellt. Die Analyse der Daten erfolgte mittels RStudio (RStudio Team (2020). RStudio: Integrated Development Environment for R. RStudio, PBC, Boston, MA, URL http://www.rstudio.com/).

Der Shapiro-Wilk-Test wurde durchgeführt, um die Studienpopulation auf Normalvereitlung zu testen. Nachfolgend wurde der nichtparametrische Mann-Whitney-U-Test beim Vergleich von 2 Gruppen verwendet, der Spearmans Rangkorrelationskoeffizient um den Zusammenhang von 2 Variablen zu berechnen. Im Fall von kategorialen Variablen wurde der Exakte Fisher-Test verwendet. Wir berechneten zudem die „odds ratio“ mit 95 % Konfidenzintervall (95 % KI). Ein *p*-Wert < 0,05 wurde als statistisch signifikant gewertet.

## Ergebnisse

### Demografie

**Figure 1:**
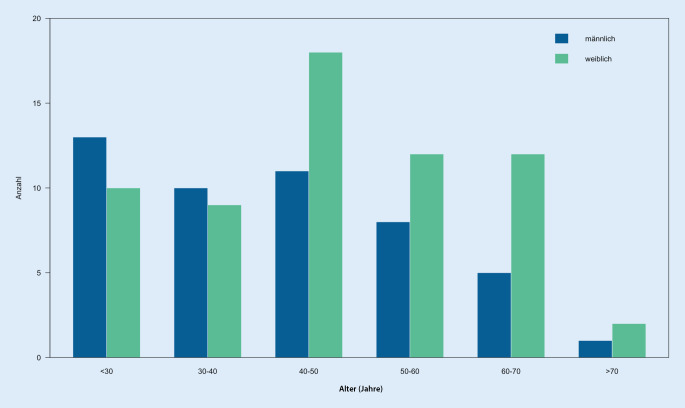

Im Zeitraum von 01.01.2003 und 31.12.2015 wurden insgesamt 111 Patienten aufgrund einer oder mehrerer Mittelfußfrakturen behandelt. Das Alter der Patienten war 45 ± 15,2 Jahre mit insgesamt 48 Männern (43 %) und 63 Frauen (57 %). Männer waren durchschnittlich 42 ± 15,3 Jahre alt und Frauen 48 ± 14,8 Jahre. Betrachtet man die Häufigkeitsverteilung des Auftretens von Mittelfußfrakturen, aufgeteilt nach Geschlecht, so findet man bei Männern unter 30 Jahren und Frauen zwischen dem 40. und 50. Lebensjahr die größte Prävalenz (Abb. 1). Über 40-jährige Patienten waren zudem 2,3 [95 % KI: 1,05–5,05] mal häufiger weiblich.

**Figure 2:**
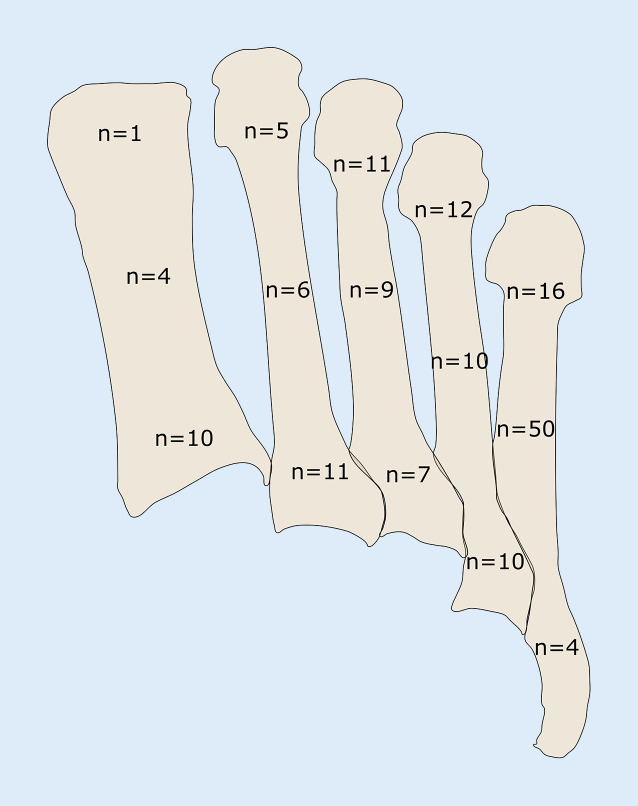

Insgesamt zogen sich die eingeschlossenen 111 Patienten 166 Mittelfußfrakturen zu. 81 Patienten (73 %) erlitten eine isolierte und 30 (27 %) multiple Mittelfußfrakturen. Am häufigsten (42 %) war der 5. Mittelfußknochen betroffen (Abb. 2), in 9 % der 1. Mittelfußknochen, der 2. in 13 %, der 3. in 16 % und der 4. in 19 % der Fälle. Bei den Mehrfachfrakturen waren in 29 von 30 Fällen unmittelbar nebeneinander gelegene Mittelfußknochen betroffen. Am häufigsten waren 3 Mittelfußknochen frakturiert (43 %), in 40 % waren 2 Strahlen verletzt, in 13 % 4 und in 3 % 5 Mittelfußknochen. In 30 % der Fälle waren der 2. bis 4. Mittelfußknochen verletzt, gefolgt von Frakturen des 3. und 4. Mittelfußknochens (17 %).

Zwischen der Gruppe der isolierten und mehrfachen Mittelfußfrakturen fand sich kein statistisch signifikanter Unterschied hinsichtlich des Alters (*p* = 0,10) oder Geschlechts (*p* = 0,67) (Tab. 1). Bei 50 Patienten (45 %) war die linke Seite und bei 61 Patienten (55 %) die rechte Seite betroffen. Insgesamt haben sich 17 Patienten (15 %) eine weitere Fraktur auf der gleichen Seite im Bereich des Sprunggelenks oder des Fußes zugezogen. Hiervon waren bei 7 Fällen die Zehen (57 % isolierte Mittelfußfrakturen), bei 3 Patienten der Kalkaneus (67 % multiple Mittelfußfrakturen), bei 3 der Talus (67 % multiple Mittelfußfrakturen), bei 2 die distale Tibia (50 % isolierte Mittelfußfraktur) und jeweils einmal die distale Fibula (isolierte Mittelfußfraktur) und das Os naviculare (isolierte Mittelfußfraktur) involviert.

**Table 1:** 

	Isolierte Mittelfußfraktur (*n* = 81)	Multiple Mittelfußfrakturen (*n* = 30)	Signifikanz (*p*‑Wert)
Alter (Jahren)	43 ± 15,6	49 ± 13,6	0,10
Geschlecht	58 % weiblich (*n* = 47)	53 % weiblich (*n* = 16)	0,67
FAOS	88 ± 17,1	78 ± 17,7	0,046*

Die Daten sind als Median ± Standardabweichung (SD) dargestellt

*FAOS* Foot and Ankle Outcome Score

*Entspricht einem signifikanten p-Wert < 0,05

Von den 81 Patienten mit isolierter Mittelfußfraktur wurden 43 Patienten (53 %) operativ nach 5 ± 3,9 Tagen behandelt. Hiervon waren 36 C-Frakturen (84 %) und 7 A‑Frakturen (16 %). In der Gruppe der konservativ behandelten Patienten hatten 28 Patienten (74 %) eine A‑, 8 (21 %) eine C‑ und 2 (5 %) Patienten eine B‑Fraktur. Die operative Versorgung erfolgte bei 26 Patienten mittels Plattenosteosynthese, 8‑mal mit einer Schraubenosteosynthese, bei 7 Fällen mit K‑Drähten (4-mal retrograde, 3‑mal antegrade Osteosynthese), einmal mit K‑Draht in Kombination mit Plattenosteosynthese und einmal mit K‑Draht in Kombination mit einer Schraubenosteosynthese. Eine Revisionsoperation war bei keinem Patienten notwendig. In 6 der 7 minimal-invasiven Versorgungen durch intramedulläre K‑Draht-Osteosynthesen erfolgte eine regelhafte Metallentfernung nach 4 ± 0,9 Monaten. Des Weiteren wurde bei 8 Patienten aufgrund des störenden Osteosynthesematerials eine Metallentfernung nach 15 ± 8,1 Monaten durchgeführt. Alle operierten Patienten zeigten nach 12 ± 11,3 Monaten eine regelrechte knöcherne Konsolidierung.

In der Gruppe der multiplen Mittelfußfrakturen erhielten 16 Patienten (53 %) eine operative Versorgung nach 4 ± 4,1 Tagen. Das operative Verfahren war in 5 Fällen eine Plattenosteosynthese, 3‑mal eine K‑Draht mit zusätzlicher Schraubenosteosynthese, in 3 Fällen eine Schraubenosteosynthese, in 3 Patienten eine K‑Draht-Osteosynthese (2-mal retrograde und einmal antegrade Osteosynthese) und 2‑mal eine Platten- mit K‑Draht und Schraubenosteosynthese. Eine Revisionsoperation war bei 3 Patienten aufgrund eines Infekts und bei 2 Patienten wegen einer sekundären Dislokation notwendig. Bei Patienten mit Revisionsoperation waren mindestens 3 Mittelfußknochen frakturiert, wovon mindestens einer eine C‑Fraktur darstellte. Die K‑Drähte wurden bei 2 Patienten nach 2 Monaten und regelrechtem postoperativen Verlauf entfernt. Des Weiteren wurde bei 5 Patienten aufgrund des störenden Osteosynthesematerials eine Metallentfernung nach 14 ± 4,3 Monaten durchgeführt. Alle operierten Patienten zeigten nach 13 ± 12 Monaten eine regelrechte knöcherne Konsolidierung.

Der Nachbeobachtungszeitraum betrug 57 ± 34,6 Monate.

### Funktionelles Ergebnis

Patienten mit isolierter Mittelfußfraktur hatten einen FAOS von 88 ± 17,1. Im Vergleich erzielten die Patienten mit multiplen Mittelfußfrakturen einen FAOS von 78 ± 17,7 und somit ein schlechteres funktionelles Ergebnis (*p* = 0,046). Patienten mit multiplen Mittelfußfrakturen berichteten insbesondere bei den Subskalen „Symptome“ und „Steifigkeit“ über Einschränkungen.

Das Auftreten einer weiteren Fraktur am ipsilateralen Fuß oder Sprunggelenk führte bei Patienten mit isolierter Mittelfußfraktur zu einem schlechteren funktionellen Ergebnis (*p* = 0,014).

In der Gruppe der isolierten C‑Frakturen hatten operierte Patienten (*n* = 36) einen FAOS von 90 ± 14,0 und die konservativ Therapierten (*n* = 8) einen FAOS von 87 ± 22,2 (*p* = 0,52).

Im untersuchten Patientenkollektiv ergab sich keine signifikante Korrelation zwischen Alter und FAOS (*p* = 0,11).

## Diskussion

Mittelfußfrakturen gehören zu den häufigsten Verletzungen der unteren Extremität [9, 16]. Insbesondere die Verletzung mehrerer Mittelfußknochen kann zu bleibenden Einschränkungen führen und in der Literatur finden sich nur wenige Studien, welche das funktionelle Ergebnis nach konservativer und operativer Behandlung von multiplen Mittelfußfrakturen sowie Verletzungen des 1. bis 5. Strahls analysiert haben [7, 20]. Es existieren zahlreiche Studien zum Outcome nach Basisfrakturen des 5. Mittelfußknochens, diese isolierten Verletzungen wurden jedoch aus dem Studienkollektiv ausgeschlossen [1, 3, 5, 6, 11, 17, 21].

In der vorliegenden Studie wurde das funktionelle Ergebnis von 111 Patienten mit Mittelfußfraktur sowohl nach konservativer als auch operativer Behandlung analysiert. Patienten mit einer isolierten Mittelfußfraktur, welche keinen signifikanten Rotationsfehler, keine relevante Verkürzung, Gelenkstufe oder Achsabweichung aufwiesen, hatten ein gutes bis sehr gutes funktionelles Outcome nach konservativer Therapie. Isolierte C‑Frakturen zeigten sowohl nach Operation als auch konservativer Therapie ein gutes Ergebnis. Patienten mit multiplen Mittelfußfrakturen hatten hingegen ein schlechteres Ergebnis und berichteten über persistierende Bewegungseinschränkungen und Steifigkeit. Nach unserem Kenntnisstand ist dies zurzeit die größte derartige Studie mit einem selbstberichteten patientenbasierten Ergebnisfragebogen.

Die Patienten im untersuchten Studienkollektiv waren durchschnittlich 45 Jahre alt, wobei die Prävalenzen insbesondere bei jüngeren Männern und Frauen im mittleren Alter besonders hoch waren. Frauen über 40 Jahre waren signifikant häufiger von einer Mittelfußfraktur betroffen. Diese Altersverteilung mit anteilig mehr Frauen im höheren Alter ist auch in anderen Studien beschrieben [16]. Der 5. Mittelfußknochen war am häufigsten verletzt (42 %) und das Auftreten von multiplen Mittelfußfrakturen in unserem Studienkollektiv deutlich seltener (27 %). Dies ist auch so in anderen Studien berichtet worden [9, 16]. Bei den multiplen Mittelfußfrakturen waren am häufigsten der 2. bis 4. Mittelfußknochen betroffen.

Entsprechend den angegebenen Kriterien erfolgte eine operative Versorgung sowohl bei den isolierten als auch multiplen Mittelfußfrakturen in etwa der Hälfte der Patienten. Bei den isolierten Mittelfußknochen wurden v. a. C‑Frakturen operativ versorgt. Das operative Verfahren der Wahl war hier häufig eine Plattenosteosynthese. Dies entspricht dem Vorgehen anderer Studien und Übersichtsarbeiten, welche bei Mehrfragmentfrakturen mit und ohne Gelenkbeteiligung die operative Versorgung mittels einer offenen Reposition und Plattenosteosynthese empfehlen [8, 18].

In der Gruppe der isolierten Mittelfußfrakturen gab es keine Komplikationen, welche eine Revisionsoperation notwendig machten; diese Ergebnisse sind im Kontext mit der gängigen Literatur. Hier ist eine komplikationslose knöcherne Konsolidierung nach operativer Behandlung beschrieben [8, 13, 24]. Bei den multiplen Mittelfußfrakturen war hingegen in 5 Fällen eine Revisionsoperation notwendig. Bryant et al. berichteten keinerlei Komplikationen nach Plattenosteosynthese von Frakturen des 1. bis 4. Mittelfußknochens. Hierbei handelte es sich jedoch um eine retrospektive Studie, und es waren nur in 22 Fällen mehr als ein Mittelfußknochen betroffen [8]. Bei 2 weiteren Studien, welche das postoperative Ergebnis nach K‑Draht-Osteosynthese von Mittelfußfrakturen untersuchten, wurde keine Angabe zu Revisionen gemacht [13, 24]. Alepuz et al. hingegen stellten bei multiplen Mittelfußfrakturen einen Zusammenhang fest, zwischen einem schlechten postoperativen Ergebnis und der Präsenz einer Mehrfragmentfraktur, Dislokation und schwerem Weichteiltrauma. In dieser Studie wurde jedoch keine Revisionsrate angegeben [2]. Da es nur wenige Studien über das postoperative Ergebnis von multiplen Mittelfußfrakturen gibt, lässt sich daher keine sichere Reoperationsrate zum Vergleich heranziehen [18].

Das funktionelle Outcome nach isolierten Mittelfußfrakturen war insgesamt sehr gut (FAOS = 88). In dieser Gruppe gab es keinen signifikanten Unterschied zwischen operativ und konservativ behandelten Patienten. Hier ist jedoch anzumerken, dass der Großteil der Patienten mit einer isolierten C‑Fraktur operativ behandelt wurde und sich daher nur ein tendenziell besserer FAOS bei den Operierten zeigte, ohne Signifikanzniveau zu erreichen. Grundsätzlich lässt sich festhalten, dass isolierte Mittelfußfrakturen, welche keinen signifikanten Rotationsfehler, keine relevante Verkürzung, Gelenkstufe oder Achsabweichung aufweisen, erfolgreich konservativ behandelt werden können. Isolierte C‑Frakturen zeigen nach operativer Therapie ein sehr gutes funktionelles Ergebnis. Dies ist im Einklang mit anderen Studien, welche das Outcome nach Mittelfußfrakturen untersuchten [8, 9].

Patienten mit multiplen Mittelfußfrakturen hatten im Vergleich mit der Gruppe der isolierten Frakturen einen signifikant schlechteren FAOS. Cakir et al. fanden bei der Untersuchung funktioneller Ergebnisse nach konservativer Therapie von Mittelfußfrakturen keinen signifikanten Unterschied zwischen isolierten und multiplen Mittelfußfrakturen [9]. Alepuz et al. berichteten über ein schlechtes funktionelles Ergebnis nach operativer und konservativer Versorgung von multiplen Mittelfußfrakturen, setzten jedoch bei der Evaluation keinen validierten Fragebogen ein [2]. Die operative Behandlung von multiplen Mittelfußfrakturen führte zu keinem besseren funktionellen Outcome. Hier gibt es bis dato keine Studie, welche den Einfluss der operativen Behandlung mittels eines patientenbasierten Ergebnisfragebogens bei multiplen Mittelfußfrakturen untersucht hat. Kim et al. und Zarei et al. berichteten in kleinen retrospektiven Studien lediglich über das funktionelle Ergebnis nach operativer Versorgung von multiplen Mittelfußfrakturen mittels K‑Draht-Osteosynthese [13, 24]. Hier fehlen jeweils die konservativ behandelten Vergleichsgruppen, um als Vergleich zu der vorgestellten Studie zu gelten.

In der vorliegenden Patientenkohorte resultierte kein Einfluss des Faktors „Alter“ auf das funktionelle Ergebnis. Dies ist im Einklang mit der einzig vergleichbaren Studie von Cakir et al., welche ebenfalls den Einfluss des Alters auf das funktionelle Ergebnis bei Patienten mit Mittelfußfraktur (validierter Fragebogen) analysierten und dabei ebenfalls keinen signifikanten Zusammenhang fanden [9]. In 15 % aller Patienten zeigte sich eine weitere Fraktur am ipsilateralen Sprunggelenk oder Fuß. Am häufigsten war eine begleitende Verletzung der Fußzehen. Das Auftreten einer weiteren Fraktur an der betroffenen unteren Extremität führte bei Patienten mit isolierter Mittelfußfraktur zu einem signifikant schlechteren FAOS. Es ist zwar beschrieben, dass Mittelfußfrakturen in Kombination mit weiteren Frakturen auftreten können, jedoch gibt es keine Studie, welche den Einfluss von Begleitverletzungen auf das funktionelle Outcome untersucht hat [2]. In anderen Studien, welche das Outcome von Mittelfußfrakturen untersucht haben, wurden Patienten mit Begleitverletzungen oftmals aus der Analyse ausgeschlossen [4, 6, 15].

### Limitationen

Die vorliegende Studie weist aufgrund des retrospektiven Designs Limitationen auf, welche bei der Interpretation der Daten zu beachten sind. Es erfolgte keine Randomisierung der Patienten mit Indikation zur operativen Therapie auf eine Interventions- (Op.) und Vergleichsgruppe (konservative Therapie). Folglich wurden Patienten mit einer dislozierten, mehrfragmentären Mittelfußfraktur in der Regel operativ versorgt, und ein Vergleich zwischen operativer und konservativer Therapie hinsichtlich des funktionellen Outcomes ist daher nur eingeschränkt möglich.

Mögliche Störfaktoren und unerwünschte Nebenwirkungen sind evtl. unterrepräsentiert.

## Fazit für die Praxis

- Mittelfußfrakturen treten häufig bei Männern unter 30 Jahren und Frauen zwischen dem 40. und 50. Lebensjahr auf.
- Nur weniger als ein Drittel der Frakturen betreffen mehr als einen Mittelfußknochen.
- Bei isolierten Mittelfußfrakturen mit geringer Dislokation führt die konservative Therapie und im Falle von Typ-C-Frakturen die Operation zu einem guten bis sehr guten funktionellen Ergebnis.
- Patienten mit multiplen Mittelfußfrakturen berichten über bleibende Einschränkungen, welche in einem schlechteren funktionellen Ergebnis resultieren.
- Das Auftreten einer weiteren Fraktur an der unteren Extremität auf der betroffenen Seite führt zu einem schlechteren FAOS.
